# An Innovative Approach to Medical-Legal Partnership: Unauthorized Practice of Law Reform as a Civil Justice Pathway in Patient Care

**DOI:** 10.1017/amj.2025.10068

**Published:** 2025-07

**Authors:** Cayley Balser, Stacy Rupprecht Jane, Antonio M. Coronado

**Affiliations:** Innovation for Justice, University of Arizona James E. Rogers College of Law and University of Utah David Eccles School of Business, USA

**Keywords:** engaged scholarship, access to justice, medical legal partnership, legal empowerment, legal innovation, participatory action research

## Abstract

This Article discusses the design of an innovative approach to the traditional medical-legal partnership. This potentially transformative service model proposes the use of unauthorized practice of law (UPL) reform to embed civil legal problem solving within a patient care setting. Unlike in the traditional medical-legal partnership — a service model which embeds lawyers within patient care settings to address patients’ justice needs — we explore the promise of patient advocacy through community-based justice workers (CBJWs): members of the community who are not lawyers but who have specialized legal training and authorization to provide civil legal help to those who need it most. This work is the result of a partnership between Innovation for Justice, a social justice legal innovation lab housed at both the University of Arizona James E. Rogers College of Law and the University of Utah David Eccles School of Business, and University of Utah Health. The present framework for UPL-reform-based medical-legal partnerships was developed through robust community-engaged research and design work across the 2022–23 academic year. This article discusses the research findings and proposes a framework for replication in other jurisdictions.

## I. Introduction

In the United States, there is no right to counsel for civil cases.^1^ For patients experiencing a civil legal issue, such as a debt collection lawsuit, an incident of domestic violence, or an eviction, this means that there is no guarantee of a lawyer. At the same time, national data reveals that the under-resourced nonprofit legal service sector lacks the capacity to serve many who seek its services. Indeed, low-income Americans receive inadequate or no civil legal assistance for ninety-three percent of their civil legal problems.^2^ Millions of low-income Americans seeking free civil legal help or attempting to problem solve in the courts are only at the top of the access-to-justice iceberg, a reality wherein two in three adults experience a legal problem and only nine percent of them are aware their problem is legal in nature.^3^

Unidentified and unresolved civil justice issues create health-harming justice needs. Further, research in the public health context indicates that eighty percent of health outcomes are affected by social determinants of health (SDOH), such as income, housing, employment, and family stability.^4^ SDOH often intersect with civil justice needs: a lack of economic stability can lead to debt collection and eviction; a lack of education access and quality can lead to failure to identify the risks associated with disengagement from the civil justice system, such as default judgment and wage garnishment; and a lack of social and community context can lead to isolation and the possibility of exacerbating detrimental home situations, including domestic violence. These interactions are referred to within this Article as justice-involved social, economic, and health needs.

Currently, “hospitals are a catching system for the whole of society’s problems”^5^ and field human needs beyond their designed medical function.^6^ Patients are waiting until they have no other choice but to come in for care, and they are coming in with more than physical health needs.^7^ Hospitals and health care centers are uniquely situated to offer team-based models of care that not only address a patient’s physical and mental health, but also provide assistance in problem solving justice needs. Medical-legal partnerships (MLPs) seek to address these health inequities by integrating lawyers in health care settings. This model allows legal helpers to work alongside care teams to address those patient needs that go beyond physical symptoms, such as justice-involved social, economic, and health needs.^8^ Finding its roots in the Civil Rights era and HIV/AIDS advocacy,^9^ the MLP was first formalized in 1993, when Boston Medical Center staff traced repeat pediatric asthma patients to unsanitary apartment conditions and subsequently contacted Greater Boston Legal Services for legal aid.^10^ Since this time, the number of MLPs in the United States has ballooned, with more than 300 MLPs operating nationwide as of 2017.^11^

As one group of scholars succinctly note, the MLP operates from three core principles: “(1) the social, economic, and political context in which people live has a fundamental impact on health; (2) [SDOH] often result in issues that require legal assistance; and (3) attorneys are uniquely qualified to provide this legal support.”^12^ The efficacy of traditional MLPs has been empirically demonstrated in various areas, including financial health,^13^ health provider knowledge and training,^14^ patient health and wellbeing,^15^ decreased patient stress,^16^ decreased emergency department utilization,^17^ increased preventative care for newborn children,^18^ and improved patient compliance with treatment.^19^ However, UPL restrictions — prohibiting anyone who is not a licensed attorney from providing legal advice — have created an unsustainable and inadequate monopoly on legal services.^20^ In recognition of the harm perpetuated by the existing regulatory scheme, multiple states are re-regulating the practice of law and expanding authorization beyond those who have completed a four year degree, three years of law school, passed a bar exam, and cleared a character and fitness evaluation.^21^ Utah is leading the country in the re-regulation of the legal profession, allowing for innovative service models where nonlawyers, with court approval, are permitted to provide limited-scope legal advice to those who need it.^22^ This then raises the research question: can unauthorized practice of law (UPL) replicate and/or expand those benefits through authorizing potentially more cost-effective, community-centered legal services from someone who is not a lawyer?

The core aim and benefit of the MLP model remains its community-centric design. As prior scholarship has noted, “MLPs help improve cross-sector communication and problem solving.”^23^ The growing body of research in this area points to the ways that this medical-legal problem solving has the potential to be community-responsive,^24^ preventative,^25^ and stabilizing.^26^ This Article adds to the MLP tradition of community-centered design and summarizes the methods, results, and discussion from a collaboration between Innovation for Justice (i4J) and University of Utah (U of U) Health to design and prospectively embed a UPL-reform-based civil legal service model in a health care setting. This Article will first explore the history of UPL reform in the United States before discussing the findings from the research process designing this innovative approach to medical legal partnerships. Next, this Article discusses support for this innovative model and the implications of implementation. This Article concludes with a discussion about expanding this model beyond Utah, as well as a discussion regarding the limitations of the present study and recommendations for future research.

## II. The History and Challenges of U.S. Unauthorized Practice of Law Reform

For many decades, people who are not lawyers but have specialized training have been assisting individuals navigating the civil justice system without the need for unauthorized practice of law reform.^27^ These individuals — often known as “nonlawyer” or “court navigators”^28^ — assist litigants navigating the court system without an attorney with their civil justice needs.^29^ In 2018, twenty-three court navigator programs existed in fifteen states and the District of Columbia.^30^ By 2023, there were sixteen new programs, bringing the total to thirty-nine, with at least five more in development.^31^ These programs are made up of navigators who are neither lawyers, nor court staff.^32^ These navigator programs are, however, often physically situated inside a court building and provide direct services to litigants without an attorney.^33^ There is no attorney-client privilege applicable to navigator relationships.^34^ The navigators in these programs “undertake a wide array of tasks on behalf of the [people they serve], such as helping them physically navigate the court; get practical information and referrals; or complete their court paperwork. Navigators also accompany [the people they serve] to court to provide emotional support, help answer the judge’s factual questions, or aid in resolving a matter with opposing counsel.”^35^

In 2019, Alaska Legal Services Corporation leveraged their existing community health aid network and the legal navigator movement to establish the community justice worker program, a program that trained individuals within the communities where they lived to provide help with legal problems within the bounds of Alaska’s UPL restrictions.^36^ Another example of community-based, in contrast to court-based, legal navigators is Legal Link. Legal Link is based in the Bay Area and trains people at community-based organizations in “legal first-aid,” which helps trainees “identify legal issues, surface unmet justice needs, and access legal protections” for the people that they work with.^37^ In early 2025, Legal Link had partnered with over seventy community-based organizations in the Bay Area to expand the legal ecosystem through creating “a pipeline of Community Justice Workers with knowledge of the legal system and fill[] a critical access to justice gap.”^38^ This “legal first-aid” model has been replicated successfully in both South Carolina and Oklahoma.^39^

Building off the momentum of these navigator programs, challenges to UPL restrictions have been raised in many jurisdictions, with varying degrees of success. Extant research indicates that consumers want legal services that are targeted, trustworthy, and timely.^40^ UPL reforms create opportunities for the design and launch of innovative approaches to legal service models that are targeted, trustworthy, and timely for the people who are going to use them. These nascent UPL reform mechanisms, while they vary by program and jurisdiction in their details and design choices, can be understood in three main buckets:^41^ 1) allied legal professional programs,^42^ 2) community-based justice worker programs, and 3) alternative business structures.^43^ Two of these buckets — allied legal professional programs and alternative business structures — are not designed to increase service delivery in low-income communities.^44^ This Article focuses on the bucket which does: community-based justice worker programs.

Community-based justice worker models “involve training and certifying individuals working at community-based organizations to offer legal advice and services in certain case types. These models target low-income individuals and require modification of/exemption from or waivers of UPL restrictions. Currently, existing projects like these are authorized through state supreme court Administrative Orders or the Utah Sandbox.”^45^ As of August 2024, nine community-based justice worker models have been authorized in six jurisdictions.^46^ Each of these models has made different design choices, varying as to who is eligible to become a community-based justice worker, the authorizing mechanism,^47^ who is eligible for services, and lawyer involvement.^48^ These design choices will be examined further in Section XI of this Article.

Despite the success and authorization of nine community-based justice worker models, these successes have not been without their challenges. Opposition from both the bar and the bench have frustrated reform efforts in many jurisdictions.^49^ Opponents often take a protectionist approach, with some citing the need “to prevent nonlawyers from interfering with the lawyer’s independent judgment.”^50^ Opposition to UPL reform also often highlights concerns about consumer harm.^51^ Lawyers and regulatory reform decision-makers are wary of innovative service models that require reform of UPL restrictions because they are worried that such models would harm consumers.^52^ To date, however, no meaningful framework for assessing lawyer harm to consumers has been adopted at the national level or in scholarship, thereby limiting the extent to which any true standard for harm might be applied to emergent service models. Further, there is no evidence that UPL reform to date has resulted in consumer harm.^53^ In addition to explicit opposition from and inaction of state bar associations, evaluation timelines present another challenge. Some UPL programs have been saddled with a predetermined sunset, meaning that they will be automatically shut down if no action is taken to extend them.^54^ Further, courts are navigating new territory when contemplating UPL reform and are often working with limited time and resources.^55^

The authors of this Article have been directly involved in the design and implementation of four of the nine authorized community-based justice worker models.^56^ Our experiences navigating the challenges within different regulatory landscapes across jurisdictions led us to this project: exploring what a UPL-reform-based medical-legal partnership might look like.

## III. Project Overview: Embedding Civil Justice Problem Solving in Patient Care

University of Utah Health (U of U Health), Utah’s only academic medical center, together with the University of Utah (The U), is building a new model for how a leading research institution can engage with and support communities. The U “strives to increase community engagement, improve health, equity and economic outcomes, and increase access to higher education for every Utahn.”^57^ Because Utah’s West Valley area is growing so quickly and is home to vital, vibrant, and diverse communities, the U is developing a new initiative, “U West Valley,” that will “provide improved access to world-class health care and provide high-quality education and training programs” for the West Valley City community.^58^

U of U Health’s 2025 strategic plan prioritizes advancing community and mission-driven work.^59^ To help accomplish this goal, U of U Health asked i4J to apply their design and systems thinking research methodologies to propose potential service models for embedding civil justice problem solving within patient care in West Valley. A Directed Step for this 2023 plan included evaluating models for integrating legal support services into patient care models to help address issues related to SDOH.^60^ This collaboration between i4J and U of U Health — the project that is the topic of this Article — addressed the challenge: *how might we explore innovative approaches to embedding civil justice problem solving in a health care setting?* The research team applied design and systems thinking research methodologies to propose civil justice problem solving interventions that had community support.

## IV. Using Design and Systems Thinking to Propose Interventions

University of Utah Hospital in West Valley, where this intervention will be implemented, anticipates providing care for patients from Utah’s West Valley City, Taylorsville, West Jordan, Magna, and Kearns. West Valley City is Utah’s second largest city, and its only city with a majority population made up of people of color.^61^ These localities have a combined population of almost 380,000 residents.^62^ Across these cities, residents are more diverse than Utah’s total population on average.^63^

To develop this UPL-reform-based MLP, i4J applied design and systems thinking research methodologies using a critical participatory action research (CPAR) lens^64^ to understand the current patient experience, the civil justice needs that West Valley patients are experiencing, and to explore innovative approaches to embedding civil justice problem solving in patient care. This project focused on the areas of Utah that the new West Valley hospital will serve, areas which are significantly more diverse than Salt Lake City,^65^ where most health care interventions are currently located. CPAR creates a research ethic and responsibility to actively commit resources, practice, and scholarship to “work with communities and movements to generate alternatives” to the way the current system operates.^66^ Using these methodologies, i4J has successfully designed and implemented three other UPL-reform-based initiatives in two jurisdictions — Domestic Violence Legal Advocates in Arizona, Medical Debt Legal Advocates in Utah, and Housing Stability Legal Advocates in both Arizona and Utah.^67^ While evaluation of these initiatives continues, early data in both Arizona and Utah highlight the potential and positive impact of each of i4J’s UPL-reform-based models for delivering legal services.^68^

## V. What We Learned from the West Valley Community

### A. Civil Justice Needs of the West Valley Community

Findings from initial interviews with system actors and community members^69^ indicate that justice needs in West Valley include personal safety, divorce, legal status, financial instability, and housing instability. West Valley community members reported experiencing employment problems, financial and housing instability, mental health needs, and responsibility for caring for family members. Further, West Valley community members reported that current legal services offered do not adequately meet the service needs of the West Valley community and they expressed a desire for more options. West Valley community members were asked about their justice needs in five different categories: housing, family, finance, health, and government or public benefits.^70^ Every West Valley community member who participated in the justice needs survey identified experiencing at least one justice problem. Overall, sixteen out of nineteen community members identified experiencing a financial issue, thirteen out of nineteen identified experiencing a family issue, twelve out of nineteen identified experiencing a health issue, and ten out of nineteen identified experiencing a housing issue.^71^ Only four out of nineteen community members identified an issue with government or public benefits.^72^ The summaries of these survey response findings are illustrated in Table 1.

**Table 1.** Community legal needs by issue type

Housing experiences: 10 community members
- Difficulty or problem paying rent: 6 - Homelessness: 5 - Difficulty or problem with subsidized housing: 5 - Problem or disagreement with landlord: 5 - Difficulty or problem finding housing: 5 - Unsafe living conditions: 3 - Difficulty or problem paying utilities (like gas, water, electricity, internet): 3 - Eviction: 2 - Landlord not fixing problems with rental: 0 - Other: - Problems with rent assistance: 3 - Problems with neighbors: 1 - Problems with roommates: 1
Family experiences: 13 community members
- Caring for sick or elderly relatives: 6 - Harassment or violence from current or ex-partner, or other family or household member: 5 - Marriage: 5 - Divorce or separation: 4 - Caring for grandchildren or other relatives who are younger than 18, who are not your children: 3 - Child custody problems: 3 - Child support problems: 2 - Other: - Criminality around the house: 1 - Widowed: 1
Financial experiences: 16 community members
- Worried about being able to pay your bills, debt, or loans: 14 - Problems with creditors: 7 - Unpaid bills, debt, or loans: 6 - Money taken out of paycheck or bank account for unpaid bills, debt, or loans: 4 - Difficulty or problem receiving assistance with paying bills, debt, or loans: 4 - Received papers from an attorney or the court for unpaid bills, debt, or loans: 2 - Other: - Inflation and taxes have risen but wages have not: 1 - Unable to pay anything outside of bills (i.e. cannot pay for car repair): 1 - Food is not affordable anymore: 1
Health experiences: 12 community members
- Medical debt: 9 - Difficulty paying medical bills: 6 - A problem with health insurance: 5 - A problem with Medicaid or Medicare: 5 - Difficulty signing up for insurance: 3 - Other: - Insurance prices keep rising: 1 - Lack of nearby medical treatment: 1
Government or public benefits experiences: 4 community members
- Problem with public benefits: 3 - Problem with unemployment payment: 2 - Problem with government payment: 1 - Problem with disability payment: 1 - Other: 0

### B. The Unmet Service Needs in West Valley’s Ecosystem of Care

West Valley community members and health care system actors reported that community members are often experiencing a range of issues which can present barriers to seeking health care. Health care providers need to take care of the patient’s presenting illness, but that can be “difficult to do when the patient needs attention in other areas like housing and finances.”^73^ People are experiencing a large range of issues that are difficult to address completely, especially when providers have limited time with each patient.^74^ When community members are experiencing a wide range of needs, preventive medical care is usually moved down their list of priorities.^75^ Health care providers told the research team that noncompliance with medical guidance occurs frequently, “especially if patients feel that health is not a priority for them at that time.”^76^

Further, patients get lost between steps when seeking services. Providers want to have the capacity to care for every patient individually and give the time that the patient needs.^77^ Unfortunately, most providers do not have the capacity to allow for this. Warm handoffs between providers are helpful, but that only goes so far.^78^ Interacting with patients requires relationship building and an understanding of how systems work.^79^ Building trust takes time, and when an issue is outside the scope of one provider and the patient must go to another, trust needs to be rebuilt.^80^ Care managers want a system where there is support for patients throughout the entire process, including before problem solving is needed.^81^

Additionally, challenges with referrals occur when providers must contact patients without an existing relationship. Health care providers expressed difficulty connecting when they cold-called patients with whom they do not have existing relationships.^82^ Providers are siloed, especially specialists, and often have different processes for patient intake that makes connection difficult.^83^ Patients become frustrated when they must explain things multiple times to different providers, which leads to disengagement with the system.^84^

Other challenges to communicating available resources include rural access problems, intersecting issues, and siloing of services. Rural access to resources is often reported to be more complicated than how systems present it.^85^ Often resources change because of need or staff turnover, and not all resource information is updated consistently.^86^ Many community-based organizations and health care providers indicated that 211 does a great job of keeping databases as up to date as possible, but 211 must rely on service providers to have accurate information available.^87^ Distant geographic location often makes services inaccessible, especially when there are few providers.^88^ Community-based organizations also do not want to duplicate efforts in the community.^89^ Many organizations serve specific segments of the population or focus on specialized issues.^90^ There is a hesitancy to offer more services in one location that people are already offering elsewhere.^91^ This means that people experiencing multiple issues must go to multiple service providers to address their needs. Further, service terms are not always consistent between government agencies, resource lists, and community-based organizations.^92^

Even when resources are available, a lack of knowledge about resources and issues prevents community members from accessing the resources that could help.^93^ Community-based organization staff shared that many community members do not know about all the resources that are available in their area.^94^ This is especially true for people who are new to the area. Some areas are resource scarce, and sometimes the resources that do exist are oversaturated.^95^ Community members do not always realize that there are resources for what they are experiencing.^96^ One care manager spoke about patients that “do not know that they’re eligible for these government programs,”^97^ and a community-based organization noted that people often do not know that the discrimination they are experiencing is illegal.^98^

### C. Barriers to Accessing Legal Services

Within the justice needs survey, the research team included three questions about interactions with the justice system within the past two years.^99^ Four community members indicated that they had experienced a problem involving an attorney, a lawsuit, a court, or a judge within the past two years, while fifteen had not.^100^ Three community members experienced a problem within the past two years that they thought might go to court, while sixteen did not.^101^ Five community members sought legal help, while fourteen did not.^102^

Barriers to West Valley community members seeking legal services included confusion, lack of trust, and lack of time. Community members do not trust attorneys.^103^ Community members expressed a desire for more trusted sources, especially when it comes to navigating justice issues.^104^ One community member said that mistrust of lawyers can be attributed to a lack of representation: “lawyers do not look like or understand the community.”^105^ Additionally, community members do not think that lawyers are worth the cost.^106^ One community member put it frankly: that “lawyers are for rich people.”^107^ It costs money to get records expunged, get legal advice, and file documents with the court.^108^ Community members do not think the cost of lawyers is worth it for the help that they will get, especially when it comes to housing and debt issues.^109^ They know when they cannot pay rent, and they know when they have to prioritize what bills get paid when.^110^ Adding another cost — the cost of legal services — is not worth it from their perspective. Further, community members want to feel like a person, not a number, and want to see themselves reflected in their service provider.^111^

Additionally, there are unique challenges and opportunities associated with serving the immigrant and refugee populations in West Valley that should be considered in intervention design.^112^ Care strategies should focus on early intervention, trust-building, cultural humility, and appropriate screening.^113^ Regardless of specific population or intervention design, system actors highlighted the importance of trauma-informed care.^114^

### D. Trauma-Informed Care is Critical

Seeking services can be traumatizing.^115^ Health care in general can be a traumatic experience, especially for community members who do not speak English. Regardless of primary language, people are often asked for the same information from different providers, having to tell their story again and again.^116^ One care manager expressed a desire to see more coordination among services and their providers to create a system of wraparound services where people are not retraumatized.^117^

Communities and research make clear that, because of the heightened risk of retraumatization in patient care settings, trauma-informed care should be the standard.^118^ System actors in multiple industries emphasized that trauma-informed care should be universally recognized and implemented in all patient interactions and settings.^119^ Multiple health care providers and staff at community-based organizations spoke about having “to meet somebody where they’re at” and noted that community members need someone who is on their side.^120^ A health care provider put it this way: “patients are left only with you and trusting you and you kind of become a gatekeeper.”^121^ They stressed the importance of knowing what is within their scope, and not providing services to the patient that are outside of that scope, but also the importance of connecting the patient with someone who does provide other needed services.^122^

Investing in and prioritizing trauma-informed practices in service model design is beneficial because trauma-informed practices are translatable to various care settings.^123^ Trauma-informed practices are not specific to any health care or clinical setting and are suitable for implementation in various settings.^124^ A health care system actor shared that these practices include “actually giving [patients] the time of day,” recognizing that every patient is different, and demonstrating a “willingness to listen to [the patient] as an individual and take [them] seriously.”^125^ Trying to understand cultural trauma that some patients have experienced is also important.^126^ Involving the community in the process is helpful, including by letting community members and patients themselves guide how they want to be treated.^127^ Cultural responsiveness is a trauma-informed practice.^128^ Someone who is not culturally aware or responsive can make the other person feel unintelligent or unwanted, triggering trauma responses or exacerbating existing effects of past trauma.^129^

### E. Problem Solving Strategies Should Focus on Early Intervention, Trust-Building, and Cultural Competence

Throughout our interviews, system actors across professions emphasized the importance of early intervention. System actors who work in community mental health and the courts expressed a desire for the removal of stigma for early diagnosis and treatment.^130^ It is their belief that diagnosing and treating mental health problems as early as possible can help lessen the number of interactions they have with the justice system, especially the criminal justice system.^131^ Early intervention is important in health care, generally, as well.^132^ Right now, “hospitals are a catching system for the whole of society’s problems,” which is not their designed function.^133^ Patients are waiting until they have no other choice but to come in for care.^134^ Health care providers expressed a desire for investment in patient health “upstream from health care” and from “a governmental and community perspective” that should include preschool access and long maternity and paternity leave.^135^

Early intervention in justice needs — before a human need becomes court involved — is helpful, particularly in housing and debt collection cases.^136^ One justice system actor said: “if a debt is owed to a creditor or a landlord, getting the parties to agree on payment before it turns over to an attorney and a collection agency is important.”^137^ Court system actors, in particular, emphasized the importance of intervention before debt is turned over to a collection agency.^138^ As one court system actor shared, the cost of collection has quadrupled in recent years.^139^ This is a fourfold increase in the amount a person owes on the debt once it is turned over to collections.^140^ A court system actor said that once a debt is in the hands of the collection agency, “the story is written,” and there is even less that a defendant can do once a complaint is filed.^141^ If a debt is owed to a creditor or a landlord, it is helpful if the parties are able to agree on a payment plan before collection agencies or attorneys are involved.^142^

Beyond early intervention, establishing trust is essential to meeting patient needs. A community health worker told the research team that “there’s a lot of mistrust in communities with systems,” which makes it “hard to be effective in the work that [community health workers] do, unless [the community] trusts us.”^143^ Trust can be built through intentional, proactive community engagement, and doing “what you say you’re going to do.”^144^ Trust can also be built by explaining why information is being collected, for what purpose, and how it will be used.^145^ Community members expressed discomfort to health care providers about their information being stored in their medical record and fear of being treated differently because of information that they share.^146^

Representation is important when building trust. If community members feel represented, they are more likely to trust.^147^ One health care provider noted that “it is important seeing someone like you” when seeking services.^148^ The area health care centers hosted focus groups with the community, and the overwhelming feedback was that the community wanted to see themselves in their service providers.^149^ This included race, ethnicity, and language.^150^ Having the appropriate interpreter available is also helpful in building trust.^151^ The West Valley community speaks over 100 languages; knowing the audience and being able to effectively communicate helps to build trust and show community members that their opinions and voices matter.^152^ The importance of representation was well documented in i4J’s engagement with community members throughout this project.^153^

When representation is not possible, cultural responsiveness and humility are imperative when building trust and offering services to the West Valley community.^154^ A system actor defined cultural humility as the theory that one may not ever fully know or understand someone else’s community.^155^ It is centered on listening to others and being aware that the person listening does not know everything.^156^ Understanding cultural trauma is an important aspect.^157^ One community-based organization leader said being “culturally responsive means that there is a deep rooted understanding of the circumstances that affect the individual being born and raised outside of the United States.”^158^ There is no checklist of items or behaviors that make someone culturally responsive.^159^ Speaking a language is not enough to be culturally responsive.^160^ Another community-based organization staff member explained that “so many immigrant and refugee migration journeys are multifaceted.”^161^ Caring for immigrant and refugee populations requires service providers of dominant demographics to get outside of their comfort zones, getting “to know your patients and ask them and engage” with them to best understand their needs.^162^ It is hard to make generalizations about working with populations because each patient is unique in their needs and preferences.^163^

Challenges in building trust include high levels of turnover, wariness of new builds, and performative community engagement.^164^ High turnover negatively impacts partnership, especially when building trust and sustaining relationships with the community is so important.^165^ One system actor said: “when you have that turnover, and systems do regularly, then that trust is broken, and you have to start over again, and that’s really rough.”^166^ The West Valley community, especially the Pacific Islander community, is wary of new builds coming in.^167^ The Pacific Islander community saw new builds in Hawai’i where hospitals and churches were built in communities, and noted that outsiders were then hired to work these new jobs instead of promoting from within the community.^168^ Another challenge that system actors expressed relating to the West Valley project was making sure that the voices of the community are heard and incorporated into every decision.^169^

## V. Support for a UPL-Reform-Based Service Model

The intervention favored by the community through feedback testing was a service model that trained community-based justice workers (CBJWs), people already living and working in the West Valley community. These CBJWs could be Community Health Workers (CHWs), staff from local community-based organizations, or other community members, including those with justice-problem-solving lived experience, and those pursuing workforce development. These CBJWs would be employed by and physically located within the health care setting, similar to the traditional MLP model. As designed, patients would be screened for health and justice problems through their interactions with U West Valley and connected to CBJW services for the needs identified in the screening.^170^

System actors indicated that new service models should consider patient needs regarding referrals, service provider preferences, and siloing of services.^171^ When considering who community members would like help from, community members identified case managers, social workers, community health workers, and community-based organizations.^172^ Community members and system actors expressed a desire for community health workers (CHWs) to step into the CBJW role.^173^ However, there is a capacity concern for CHWs becoming CBJWs.^174^ Current Licensed Paralegal Professional^175^ opportunity requirements present an insurmountable barrier to CHWs and community-based organizations wishing to leverage UPL reform.^176^ The CBJW program is a more enticing avenue to embed civil justice problem solving within health care settings because of the lower barriers to entry.^177^ When asked about concerns, liability was the highest concern among all categories of system actors.^178^ Supervision and oversight of CBJWs by attorneys would mitigate this concern.^179^ If implemented, community-based justice workers should be properly trained and adequately compensated for providing legal services.^180^ The research team ended this phase of the work by recommending further investigation into the design and viability of the CBJW model to University of Utah Health leadership.^181^

In Spring 2023, U of U Health leadership agreed with the recommendation and asked i4J to continue the design work on the CBJW service model. This phase of the project focused on identifying and beginning to answer questions related to the intricacies and details of what it would take to embed CBJWs in patient care. In collaboration with the Population Health Center at U of U Health, i4J created a research guide detailing how other clinics can replicate the design work for their specific clinic.^182^ This includes creating a service model blueprint, visualizing the interactions between clinic staff members, patients, and CBJWs along the patient health journey. This research guide uses the Intensive Outpatient Clinic (IOC) as a case study.

Key takeaways from the continued work on this project in Spring 2023 include:^183^

- Each clinic setting is unique, and embedding CBJWs should align with the clinic’s mission, vision, and values;
- Spending time creating a service model blueprint is a valuable strategy for identifying unknowns and working towards resolving those before onboarding any CBJWs; and
- At the Intensive Outpatient Clinic, it is more feasible for a CBJW to be a full-time job, as opposed to one created by upskilling someone already working at the clinic. This may be consistent across other clinic settings, but further research should be done for each clinic setting before making that determination.

## XI. Implications of Implementing an Innovative MLP

Prior literature at the intersection of community health, law, and policy has sought to engage with the global movement toward *legal empowerment*: a participatory justice framework that aims to empower nonlawyers to “understand, use, and shape the law.”^184^ Within the past decade, programs all over the world have increasingly contributed toward this aim by deploying strategies of community mobilization, legal literacy, community-based paralegals, and right to information laws to expand access to public services for historically marginalized and underserved communities.^185^ In the health context, particularly, this work has “focus[ed] on the interface of people and the state.”^186^ As one meta-analysis of legal empowerment programs in low- and middle-income countries reports, several key elements drive these legal empowerment efforts in the health advocacy space, including: 1) a focus on raising awareness of individuals’ health rights; 2) efforts to collectively mobilize communities to tackle shortcomings of the current system; 3) emphasizing the importance of documentation of rights violations; and 4) “the training, deployment, and support of paralegals to help individuals to navigate the grievance redress process.”^187^

Evidence for the international adoption of a legal empowerment framework can be found in the establishment of the global Commission on Legal Empowerment of the Poor^188^ and the UN General Assembly’s 2008 resolution underscoring best practices for legal empowerment.^189^ Likewise, in the United States, a growing collective of scholars, practitioners, and activists have embraced this paradigm for legal intervention and have championed its potential to advance community-centered justice.^190^ Of great note are the ways that this movement toward legal empowerment is consonant with existing U.S. efforts to adopt *participatory justice* — or the intentional involvement of communities “in building voice and agency regarding how they are protected from crime and victimization”^191^ — and to *democratize* the law.^192^ Thus, under the banner of many names, community-engaged initiatives to radically change how nonlawyers “know and use the law” continue to gain traction.^193^

As the present service model underscores, radically new legal service models that can complement traditional legal services are needed to serve the unmet justice needs of low-income communities. The health care sector provides a unique opportunity, particularly in Utah, to expand the categories of people who can provide legal help to those who need it and do so earlier, before a socio-economic problem becomes a legal problem. As previously mentioned, Utah is leading the nation in re-regulating the practice of law to permit new types of legal service models.^194^

As explored across this Article, the critical participatory action research of the present study has led to the design of the CBJW Initiative: an MLP that provides UPL-reform-based legal assistance. By leveraging UPL reform opportunities available in Utah, under-represented and historically marginalized populations could benefit from and become community-based justice workers. CBJWs are advocates with legal training but not a J.D., who “would not be limited to legal routes to obtain solutions; rather, [they] would be focused on helping people understand their options and resolve their substantive problems.”^195^

This intervention was designed in direct response to community feedback and is readily adaptable to various clinical settings.^196^ As members of this research team have noted before, a primary benefit of this model includes the potential for CBJWs to be people already living and working in the community. This creates job opportunities within the communities that need civil justice problem solving help. In contrast, other interventions — including traditional MLPs — bring in “outsiders” that the community says they do not want and do not trust. This is congruent with existing access to justice research, finding that people experiencing justice needs appreciate legal problem-solving assistance that is timely, targeted, and trustworthy.^197^ Further, this service model leverages the deep lived and learned knowledge of community needs that already exist in the people that live and work there. West Valley is a diverse community that speaks over 100 languages, where current help-seeking barriers include lack of language and cultural understanding between provider and patient. Training CBJWs that are already embedded within the community and have the cultural and linguistic aptitude significantly mitigates these barriers. Further, U of U Health is aware of the potential displacement that the hospital could cause in the West Valley community and are working towards mitigation efforts, including the creation of employment opportunities for those already residing in West Valley.^198^ Implementation and evaluation of this proposed intervention will contribute necessary data to accurately measure whether benefits provided by a CBJW are commensurate with those provided by a traditional MLP.

## XII. Expanding this Innovative Model Beyond Utah

As more states contemplate reforming their UPL restrictions, it is important to note that there is no one CBJW program or approach that will solve the access to justice crisis on its own. As other jurisdictions contemplate innovative service models, the authors suggest a non-exhaustive list of seven questions to reflect on when making design choices.^199^ First, what unmet community needs would the service model address? As mentioned previously, consumers want services that are trusted, targeted, and timely.^200^ Innovative service models that have been successfully implemented have engaged in early-stage research to define their target populations, needs, and goals. Positioning these findings as the guide in legal design choices helps to define success and ensure that any proposed model meets the needs as defined by the community. Second, who is positioned and trusted in the community to meet those needs? A proposed service model could have all the right ideas, but if the people identified as potential new service providers do not want to provide the service, or are not trusted by the community, then the service cannot launch. Examples of trusted members of the community who could provide services include staff at community-based organizations, legal aid, or other community actors. To find who these trusted people are and to understand what their capacity for new services includes, ask the community who they already go to for support, learn more about the resources available to these trusted people, and explore whether and how they want to become involved in innovative service models.

Third, because of the great variation in UPL restrictions by jurisdiction,^201^ it is important to determine whether the service model will require UPL reform and whether that is something feasible in your jurisdiction. For example, legal navigator programs in the courts and community have been providing legal help for decades within existing UPL restrictions. An innovative MLP model could be training lay people in “legal first aid” in a health care setting instead of fully crossing into a UPL reform regulatory environment. This may be a more feasible form of this service model in jurisdictions that are hostile to reform efforts. In other jurisdictions where there has been successful reform efforts and implementation, it is important to understand the regulatory reform mechanism that would make the service model possible.

Understanding whether a reform mechanism is needed then leads to the fourth question: who will mentor and train these new legal workers? Examples from currently implemented community-based justice worker models include lawyers at a nonprofit, lawyers at legal aid, a legal education institution such as one housed at a University, or judges or other court personnel.^202^ When considering who would mentor and train new legal workers, it is important to find the opportunity space in the overlap between who new legal workers want to learn from and who has the capacity to dedicate the necessary time and intention to training and mentoring.

Fifth, who will credential the community-based justice worker? Research shows that community members are more likely to trust legal help from someone who is trusted in their community and who has been credentialed to provide legal help.^203^ Successfully implemented community-based justice worker programs have answered this question in various ways, including through credentialing by the state supreme court after an exam, application and authorization through a regulatory sandbox, or supervision by legal aid.^204^ In addition to being an impetus for community trust in the legal worker, credentialing also helps legitimize their role in civil justice problem solving in court settings and assures civil legal system actors that community-based justice workers are working within the scope of their authorization.

Sixth, after contemplating credentialing, jurisdictions should explore whether the service model needs insurance. Some questions to ask when making this design decision include considering who will be responsible for the insurance: the justice worker themselves, the organization where they work, or the supervising entity (if one exists)? Additionally, will insurance, or lack thereof, impact community eligibility for services? For example, federally funded legal services have specific income requirements for services and exclude undocumented and incarcerated community members.^205^

The final design decision that must be made pertains to the scope of service. This should be determined by looking to the community’s unmet needs as well as the capacity that the trusted community members have to offer help. The scope of services could range from tasks that do not require UPL reform similar to those provided by legal navigators all the way to representation in court. When making decisions about the scope of service, it is important to determine priorities. This means finding the right balance for the program and jurisdiction between short training for simple, high-need areas and longer training for more complex problems. Jurisdictions should take into account both community need and what the authorizing entity will tolerate, especially if UPL reform is necessary.

Cutting across each of the design choices contemplated above is a common question of evaluation. Namely, in developing new service models for the delivery of legal help, do prevailing models for service evaluation remain reliable, meaningful, and replicable? As these authors and fellow researcher-scholars recognize, answering this question requires that we adopt “a people-centered perspective” as to the goals of a given legal service innovation and that we identify new “measures of justice.”^206^ This necessarily includes the evaluation of community-based justice worker models — particularly ones in the patient care context. Prior literature underscores the importance of this people-centered approach to evaluation in the patient care setting, especially given the ways that patient-reported outcome measures can contribute to “survey fatigue,” in which the inundation of a service recipient with requests for data that may result in patients “fill[ing] out questionnaires as quickly as possible without adequate thought, respond[ing] to only some of the queries, or ignor[ing] questionnaires altogether.”^207^ With care and community top of mind, these authors join fellow data practitioners in advocating for a “Do No Harm” approach to the evaluation of new service models,^208^ wherein data equity and community-, context-, and power-conscious evaluation metrics are prioritized.^209^ As discussed at greater length in the section that follows, evaluation tools for new service models should receive the same level of intentional and community-responsive design as the underlying service model.

As these authors have learned through designing and implementing multiple community-based justice worker models, it is important to learn about concerns from all system actors early in order to address them thoroughly prior to seeking authorization and planning for implementation. The design and systems thinking research methodologies explored in this Article can be adapted and used in other jurisdictions and in any setting to determine what the best fit for an innovative service model might be. Additionally, further steps can be taken to determine design details through the creation of a service model blueprint.^210^

## XIII. Limitations and Recommended Additional Research

Limitations of the study discussed in this Article situate this topic well for future research. First, due to resource limitations, only individuals who speak English were included in survey and interview participation. This limits the generalizability of results to members of the West Valley community who are not proficient in English, as they are not directly represented in this research. Given our inability to translate this study into other languages, we interviewed bi- and multilingual health care providers, who acted as a proxy for community members not proficient in English. Second, this project focused specifically on the West Valley community in Utah; further research is needed to determine whether any identified insights are applicable to other jurisdictions and communities. In addition to the lack of generalizability of this study, throughout the research process the research team determined that further community-engaged theorizing and research is needed prior to service model implementation, as demonstrated by the following queries.

### A. Is there an opportunity in Utah for providers to receive Medicaid reimbursement when addressing health-harming justice needs?

When creating the CBJW service model blueprint, the research team discussed with the Population Health Center team whether it was possible to expedite patient onboarding at the IOC if a patient was experiencing a justice need. At this point, such a configuration is not possible, because U Health IOC funding comes through Medicaid. At this time, Medicaid reimbursement is not allowed for health-harming justice needs for this clinic. That authorization would need to occur prior to reconfiguring the patient onboarding used at the IOC. During this research process, the research team learned of a medical-legal partnership (MLP) in another jurisdiction that has succeeded in getting Medicaid reimbursement for health-harming justice needs. The research team is in contact with leadership in that jurisdiction and plans to update the Population Health Center team with more information and details as they are learned.

### B. What types of justice needs are experienced by the IOC patient population, and what is their prevalence?

At this point, the exact types and rates of civil justice needs experienced by the IOC patient population are unknown. To accurately and effectively answer this question, clinics should conduct a justice needs survey or assessment. Because of literacy concerns and a lack of technology accessibility for some patients, an asynchronous online or paper survey is likely not well suited for this patient population. Synchronous online or paper surveys might be effective if IOC staff can ask patients questions and help them fill out the survey. This specific patient population might need additional follow up for clarification, but it would be workable for gathering a baseline understanding.

### C. How can CBJWs impact be scaled and optimized?

In order to answer this question, we suggest several areas of additional community-engaged research. First, we believe it is of great importance that innovators in this space develop a baseline dataset and framework for evaluating the potential impact of patient-based legal advocacy by CBJWs. Second, we suggest that future applied research and scholarship-in-action conduct an initial test run of what this service model would look like in order to iterate. At this point, a CBJW-based service model for patient legal care is still a theoretical concept. Implementation of this service model would likely identify challenges and opportunities yet unknown. This might include a deeper investigation of community support for possible UPL-reform-based service models and focused examination of community reluctance to a student-based clinical model. Lastly, we anticipate that authorization of this service model may present barriers or opportunities for further exploration. Because this service model has not yet been authorized by the Utah Supreme Court, it is unknown what the exact scope and capacity of CBJWs will be from the Court’s perspective.

### D. Are CHWs a good fit for becoming CBJWs?

Throughout this project, CHWs came up repeatedly as a good fit for a CBJW role. However, more engagement with CHWs should occur to ensure that this is something they want and have capacity to do. CHW leadership has indicated that CHWs would likely be interested in this additional training, but CHWs themselves have not been asked.^211^ Involving CHWs would be especially important when creating any certification materials, as this would help ensure that the time and effort commitments match with CHW capacity.

In this research, it was difficult to simplify complex patient experiences into a generalizable two-dimension blueprint, especially with a clinic as patient-centered as the IOC. While the service model blueprint deliverable from this case study contains state-, place-, and clinic-specific contours, it is likely and expected that, as this research process is replicated in other patient care settings, service model blueprints will vary. Each clinic that undertakes this process should end their research with a service model blueprint that is unique to their setting, staff, and patient population.

i4J’s evaluation of similar nonlawyer community-based justice worker initiatives point to the promise and potential of the CBJW model for care:

- **i4J Data Collection Generally** - With three UPL-reform projects actively operating in Arizona and Utah, i4J is currently engaged in quantitative and qualitative evaluations of the effectiveness of these models. i4J employs a range of assessment metrics and works in regular collaboration with its partner community-based organizations, launched community-based justice workers, and system actors to both define and be responsive to community measures of “success” in the work of justice-making.^212^ Beginning with i4J’s Fall 2024 multi-state cohort of community-based justice workers, we received clearance from the Institutional Review Board (IRB) to more systematically evaluate and report on our suite of UPL-reform projects.^213^ Active and in-training community-based justice workers complete regular, required surveys that assess their legal empowerment course experience and delivery of free legal help across several dimensions.
- **i4J Data Collection in DV Justice Work** - In Arizona’s domestic violence advocacy space, i4J’s Domestic Violence Legal Advocate (DVLA) Initiative has undergone robust evaluation through a randomized control trial (RCT), assessing in both quantitative and qualitative terms the procedural justice and court-based outcomes of community clients who receive limited-scope services from i4J’s launched DVLAs.^214^ To date, i4J and its DVLA community partner have not received a single complaint or report of consumer harm resulting from the community-based justice workers’ provision of legal services. In fact, qualitative evaluation has demonstrated overwhelmingly positive feedback from community clients.^215^ Descriptive statistics demonstrate that—within the first 5 months of statewide data collection and among reported service data—DVLAs’ legal advocacy was associated with the issuance of 4 child support orders; 8 legal decision-making/parenting time orders; 2 legal separation/dissolution orders; 10 orders of protection; 2 paternity action orders; 7 temporary orders; 3 waivers of mediation; and 1 court proceeding avoided through pre-court legal help.^216^ Additionally, within the first 5 months of statewide data and among reported service data, DVLAs’ legal services were associated with $3,900 of child support awards for DV survivors and $1,100 in filing fees waived.
- **i4J Data Collection in Medical Debt Justice Work** - Likewise, in Utah, i4J’s Medical Debt Legal Advocates (MDLA) adhere to the regular, monthly reporting requirements of the state’s regulatory Sandbox.^217^ This includes continuous reporting, through our community partner, on the number of unique clients, the types of services sought, the length of services provided, as well as the financial and legal outcomes of a given community client’s case.^218^ Like in Arizona, no complaints or reports of consumer harm have been recorded in association with i4J’s training of Medical Debt Legal Advocates (MDLAs) in Utah.^219^ Between May 2023 and April 2024, active Medical Debt Legal Advocates provided free limited-scope legal services to 234 unique clients experiencing medical debt.^220^ These services correspond with a projected $531,595 in net positive financial outcomes for clients and an additional $54,557 in finalized medical debt reduction. Taken together, these MDLA services were associated with over half a million dollars in either pending or confirmed medical debt relief for Utahns.^221^
- **i4J Data Collection in Housing Justice Work** - Upon their Fall 2024 launch, i4J’s Housing Stability Legal Advocates (HSLAs) have now joined this framework for reporting and work with i4J to record the client- and organization-level outcomes of their work. Between August 1, 2024, and January 31, 2025, statewide data collection on the HSLA Initiative in Arizona was associated with 80 hours of free legal help to 21 unduplicated tenants and individuals at risk of eviction across Maricopa and Pima Counties.^222^ This included 3 instances of in-court legal advocacy before 2 Maricopa County Justice Courts within the reporting period.^223^
- **Building New Metrics in Justice Work** - Across i4J’s initiatives, assessment efforts aim to pair the numeric and anecdotal outcomes of justice workers’ limited-scope legal advocacy to best capture the proven impact of legal work by nonlawyers, without overburdening the individual advocate or recipient of services. Regular data collection from i4J’s suite of UPL-reform projects occurs on a monthly basis and in coordination with all participating advocates. Evaluation metrics include county-level, case-level, and month-level summaries of both the legal and holistic care outcomes associated with an advocate’s limited-scope legal services. This replicable model for community-designed and-driven legal empowerment presents a novel pathway for expanding access to legal power for historically and currently disinvested communities as they navigate civil justice needs. In the health care context, this necessarily includes the opportunity to rethink the traditional model for pairing patient care with legal services.^224^

As our work continues to design and implement the UPL-reform-based MLP explored in this piece,^225^ it is important to note that the CBJW service model has not yet been approved by the Utah Supreme Court and no iteration of the model should be implemented prior to receiving Court authorization. If implemented prior to receiving authorization, CBJWs would run the risk of violating UPL restrictions.^226^

## XIII. Conclusion

At a time where nearly four-in-ten Americans do not have confidence in their state court systems and where only twenty-five percent have a positive view of the legal profession,^227^ the urgency of building local and community-based forms of justice-making has never been more urgent. Particularly for individuals navigating linked legal and medical systems, advancing models of community-based care that provide holistic human services — including bundled legal and medical services — is of life-altering importance. As explored across this project, the future and potential of the medical-legal partnership are foregrounded by much-needed reorientations of the legal and medical professions. In looking to the initial findings of this critical participatory action research in community and with community members, this Article amplifies the voices of the West Valley, Utah, community in identifying the need for community-based justice work in tending to the myriad and intersecting legal, medical, and human needs of these individuals. The research implications of these initial findings and community-identified needs cannot be overstated: CBJWs have the potential to be simultaneously proximate to and embedded in the care networks of their neighbors’ legal-medical needs. In every sense, the CBJW model aims to recontextualize and re-situate the MLP in the needs, the people, the staff, the places, and the lived realities of the communities in which it exists.

In drawing upon the existing literatures on U.S. medical-legal partnerships and the global movement toward legal empowerment, the present project introduces a replicable framework for embedding the emerging civil justice problem solving of UPL reform initiatives within the patient care context. The CBJW, as a distinct articulation of community care and legal power, can be seen as a marked departure from both the traditional and MLP-type models for aid in legal-medical contexts. As both members of and workers in service of their communities, CBJWs are uniquely positioned to understand, be responsive to, and tailor services in recognition of an individual client-community member’s lived experiences. Unlike the service models and prevailing paradigms before it, community-based justice work invites all of us to dream of collective futures premised on collaborative, trauma-informed, and community-driven networks of care.

## Footnotes

1 Tonya L. Brito, *The Right to Civil Council*, Daedalus, Winter 2019, at 56, 56.

2 Legal Servs. Corp., The Justice Gap: The Unmet Civil Legal Needs of Low-Income Americans 19, 48 (2022), https://lsc-live.app.box.com/s/xl2v2uraiotbbzrhuwtjlgi0emp3myz1.

3 Rebecca L. Sandefur, Am. Bar Found., Accessing Justice in the Contemporary USA: Findings from the Community Needs and Services Study 3–4, 14, 16 (2014), http://www.americanbarfoundation.org/uploads/cms/documents/sandefur_accessing_justice_in_the_contemporary_usa._aug._2014.pdf (reporting results of surveying a random sample of 668 adults from a typical middle-sized city in the Midwestern United States about certain “situations [they] may have experienced”); *see also* Pew Charitable Trusts, *How to Make Civil Courts More Open, Effective, and Equitable*, Pew Trusts 2 (Dec. 13, 2023), https://www.pewtrusts.org/en/research-and-analysis/reports/2023/09/how-to-make-civil-courts-more-open-effective-and-equitable.

4 Melody L. Greer et al., *Social Determinants of Health Data Quality at Different Levels of Geographic Detail*, 302 Stud. Health Tech. & Informatics 217, 217–218 (2023).

5 Interview with Health Care Professional (Fall 2022) (transcript on file with authors).

6
*See, e.g.*, Aviva J. Musicus et al., *Implementation of a Rooftop Farm Integrated with a Teaching Kitchen and Preventive Food Pantry in a Hospital Setting*, 109 Am. J. Pub. Health 1119 (2019).

7
*See, e.g.*, John Haupert, *Hospitals’ Community Benefits Extend Far Beyond ‘Just’ Medical Care*, Chief Healthcare Exec. (Nov. 22, 2023), https://www.chiefhealthcareexecutive.com/view/hospitals-community-benefits-extend-far-beyond-just-medical-care-viewpoint.

8 Caitlin Murphy, Nat’l Ctr. for Med.-Legal P’ship, Making the Case for Medical-Legal Partnerships: An Updated Review of the Evidence, 2013-2020, at 1, 3–4 (2020), https://medical-legalpartnership.org/wp-content/uploads/2020/10/MLP-Literature-Review-2013-2020.pdf; *see generally* Anne M. Ryan et al., *Pilot Study of Impact of Medical-Legal Partnership Services on Patients’ Perceived Stress and Wellbeing*, 23 J. Health Care Poor & Underserved 1536 (2012); Faisal S. Malik et al., *Improving the Care of Youth With Type 1 Diabetes with a Novel Medical-Legal Community Intervention: The Diabetes Community Care Ambassador Program*, 44 Diabetes Educ. 168 (2018); Jeffrey Martin, Audrey Martin, Catherine Schultz & Megan Sandel, *Embedding Civil Legal Aid Services in Care for High-Utilizing Patients Using Medical-Legal Partnership*, Health Affs. Forefront: Innovations Care Delivery (Apr. 22, 2015), https://www.healthaffairs.org/content/forefront/embedding-civil-legal-aid-services-care-high-utilizing-patients-using-medical-legal.

9
*See, e.g.*, Elizabeth Tobin-Tyler & Joel B. Teitelbaum, *Medical-Legal Partnership: A Powerful Tool for Public Health and Health Justice*, 134 Pub. Health Reps. 201, 201 (2019).

10 Joel Teitelbaum & Ellen Lawton, *The Roots and Branches of the Medical-Legal Partnership Approach to Health: from Collegiality to Civil Rights to Health Equity*, 17 Yale J. Health Pol’y L. & Ethics 343, 357–58 (2017).

11
*See* Marsha Regenstein, Jennifer Trott & Alanna Williamson, Nat’l Ctr. for Med.-Legal P’ship, The State of the Medical-Legal Partnership Field: Findings from the 2016 National Center for Medical-Legal Partnership Surveys 4 (2017), https://medical-legalpartnership.org/wp-content/uploads/2017/07/2016-MLP-Survey-Report.pdf.

12 Omar Martinez, Miguel Munoz-Laboy & Robin Davison, *Medical-Legal Partnerships: An Integrated Approach to Advance Health Equity and Improve Health Outcomes for People Living with HIV*, 4 Frontiers Reprod. Health art. no. 871101, at 2 (2022).

13 One MLP working with cancer patients was able to generate nearly $1 million through resolving denied benefit claims. Tishra Beeson, Brittany Dawn Mcallister & Marsha Regenstein, Nat’l Ctr. Medical Legal Partnership, Making the Case for Medical-Legal Partnerships: A Review of the Evidence 5 (2013), https://www.medical-legalpartnership.org/wp-content/uploads/2014/03/Medical-Legal-Partnership-Literature-Review-February-2013.pdf. Another MLP in Illinois saw a 319% return on investment, relieving $4 million in health care debt and claiming $2 million in additional Social Security benefits for participants. *Id.*

14 One study found that residents in clinics with MLPs were more confident in their personal understanding of the SDOH, were found to screen more frequently, and were more confident in their knowledge of a patient’s rights and access to public benefits. The results of the study were not statistically significant, but residents were much more likely to ask patients about their social history and housing situation. *Id.* at 6.

15 In the past few decades, researchers found that a MLP working with pregnant mothers improved prenatal health behaviors, pregnancy outcomes, lower rates of child maltreatment, and higher rates of maternal employment for program participants. Likewise, a 2012 study found a 91% reduction in emergency department visits and hospitalizations for adults with asthma, and 91% of participants also dropped 2 or more classes in asthma severity for individuals who participated in an asthma abatement related MLP. *Id.*

16 One study found that a MLP was able to reduce perceived patient stress by an average of 31% (as measured by PSS-10) and saw decreases in legal concerns and perceived wellbeing of 82% between pre- and post-intervention (as measured by a modified MyCAW Profile Score where patients were specifically asked about legal concerns). Ryan et al., *supra* note 8, at 1538, 1539, 1544.

17 Research at Pennsylvania’s Lancaster General Hospital found that the MLP model was associated with 50% decreases in emergency department and inpatient utilization and 45% decrease in overall medical costs. Martin, Martin, Schultz & Sandel, *supra* note 8.

18 Families who received care through an MLP model were more likely to have correct immunizations (77% vs 63%), more likely to have 5 or more routine preventative care visits by age 1 (78% vs 67%), and less likely to have visited the emergency room by 6 months (37% vs 50%). Robert Sege et al., *Medical-Legal Strategies to Improve Infant Health Care: A Randomized Trial*, 136 Pediatrics 97, 97 (2015).

19 As highlighted by the National Center for Medical-Legal Partnership’s 2017 *State of the Medical-Legal Partnership Field*, “[o]ver half of [surveyed] health care organizations report that clinicians have noted impacts as a result of their participation in MLPs. Sixty-six percent of health care organizations say that clinicians report improved patient health outcomes, 39 percent report improved patient compliance with medical treatment, and 23 percent report the ability to perform at ‘top of license.’” Nat’l Ctr. Medical Legal Partnership, The State of the Medical-Legal Partnership Field: Findings from the 2016 National Center for Medical-Legal Partnership Surveys 5 (2017).

20
*See, e.g.*, Laurel A. Rigertas, *The Legal Profession’s Monopoly: Failing to Protect Consumers*, 82 Fordham L. Rev. 2683, 2696 (2014); Alex J. Hurder, *Nonlawyer Legal Assistance and Access to Justice*, 62 Fordham L. Rev. 2241, 2242 (1999); Michele Cotton, *Improving Access to Justice by Enforcing the Free* *Speech Clause*, 83 Brook. L. Rev. 111, 150 (2017); *see generally* Deborah L. Rhode & Lucy Buford Ricca, *Protecting the Profession or the Public? Rethinking Unauthorized-Practice Enforcement*, 82 Fordham L. Rev. 2588 (2014).

21 Cayley Balser et al., *Leveraging Unauthorized Practice of Law Reform to Advance Access to Justice*, 18 L.J. Soc. Just. 66, 67 (2024).

22 Deno G. Himonas & Tyler J. Hubbard, *Democratizing the Rule of Law*, 16 Stan. J. C.R. & C.L. 261 (2020). In Utah, organizations can apply to the Regulatory Sandbox through the Office of Legal Service Innovations for authorization to provide legal services by people who are not lawyers. *Id.* at 69–75. This UPL reform mechanism is the vehicle that would authorize the intervention proposed in this article. *See also Utah Office of Legal Services Innovation*, Utah Sup. Ct., https://utahinnovationoffice.org/ [https://perma.cc/B46T-RRXX] (last visited June 10, 2024).

23 Tobin-Tyler & Teitelbaum, *supra* note 9, at 203.

24
*See, e.g.*, Martinez, Munoz-Laboy & Davison, *supra* note 12, at 6.

25 Murphy, *supra* note 8, at 3.

26
*Id.* at 4.

27 “Because the movement to make use of nonlawyers as navigators more widely in court settings is relatively new, many programs have been established recently. More than half of the programs have started since 2014, with many of those beginning operation in just the last two years. The remaining programs were founded since 2002, except for one program that dates back to 1981.” Mary McClymont, Just. Lab at Georgetown L. Ctr., Nonlawyer Navigators in State Courts: An Emerging Consensus 14 (2019), https://www.law.georgetown.edu/tech-institute/wp-content/uploads/sites/42/2023/06/Nonlawyer-Navigators-in-State-Courts.pdf.

28 Navigators are embedded within court systems, while not all navigators are embedded within the court. Id.; Mary McClymont, Just. Lab at Georgetown L. Ctr., Nonlawyer Navigators in State Courts: Part II — An Update 5 (2023), https://www.law.georgetown.edu/tech-institute/wp-content/uploads/sites/42/2024/05/Nonlawyer-Navigators-in-State-Courts-Update.pdf.

29
*Id.*

30
*Id.*

31
*Id.* at 4.

32
*Id.* at 5.

33
*Id.*

34
*Id.* at 6.

35 Mary McClymont, Just. Lab at Georgetown L. Ctr., Nonlawyer Navigators in State Courts: An Emerging Consensus 14 (2019), https://www.law.georgetown.edu/tech-institute/wp-content/uploads/sites/42/2023/06/Nonlawyer-Navigators-in-State-Courts.pdf.

36
*Community Justice Worker Program*, Alaska Legal Servs. Corp., https://www.alsc-law.org/community-justice-worker-program/ [https://perma.cc/6AAT-VF2A] (last visited Sept. 4, 2024).

37
*Our Mission + Model*, Legal Link, https://legallink.org/our-mission-model [https://perma.cc/BC2N-Y55Q] (last visited Mar. 20, 2025).

38
*Id.*

39 James Teufel, Evaluation of Legal First Aid: Community Participant Experiences, Satisfaction, and Utilization (Mar. 27, 2024) (unpublished manuscript), https://legallink.org/wp-content/uploads/2024/10/J.-Teufel-Legal-Link-3-State-Data-Evaluation-Report-3.27.2024-.pdf; Tanina Rostain & James Teufel, *Measures of Justice: Researching and Evaluating Lay Legal Assistance Programs*, 51 Fordham Urb. L.J. 1481, 1489 (2024).

40 Rebecca Sandefur, *Bridging the Gap: Rethinking Outreach for Greater Access to Justice*, 37 U. Ark. Little Rock L. Rev. 721, 723 (2015).

41
*See Regulatory Models*, Inst. for the Advancement of the Am. Legal Sys., https://iaals.du.edu/projects/unlocking-legal-regulation/regulatory-models [https://perma.cc/L3GP-VSPC] (last visited Mar. 20, 2025) (providing this three-part taxonomy, along with the policy tool of “regulatory sandboxes”, and giving numerous examples of implementations).

42 Allied legal professional programs (ALPs) operate by licensing paraprofessionals to provide legal services in limited situations. For more information about ALP programs, see generally Michael Houlberg & Janet Drobinske, *Inst. for the Advancement of the Am. Legal Sys., The Landscape of Allied Legal Professional Programs in the United States* (2022), https://iaals.du.edu/sites/default/files/documents/publications/landscape_allied_legal_professionals.pdf.

43 “Alternative business structures” are business structures that do not conform to the ABA Model Rule of Professional Conduct’s prohibitions against lawyers or law firms engaging in fee sharing with non-lawyers or forming a partnership with a nonlawyer if any of the partnership’s activities consist of the practice of law, and against the practice of law in any for-profit corporation where “a nonlawyer is a director or officer, can control the attorney’s professional judgment, or has an ownership interest in the enterprise.” Jayne R. Reardon, *Alternative Business Structures: Good for the Public, Good for the Lawyers*, 7 St. Mary’s J. on Legal Malpractice & Ethics 304, 308–09 (2017). Alternative business structures will not be discussed further in this Article because that model of reform does not typically involve direct service provision by people with specialized legal training who are not attorneys.

44 Because allied legal professional (ALP) programs are market-based models that generally upskill paralegals or those with legal service backgrounds to be able to provide legal services without attorney supervision in certain case areas, they are not designed to increase service delivery for low-income community members. *See Regulatory Models*, *supra* note 41; Balser et al., *supra* note 21, at 106.

45
*Id.*

46
*US Community-Based Justice Worker Programs Information Chart*, Innovation for Just., https://docs.google.com/spreadsheets/d/1yJIHSRy9k-l8wq8D6QRpqAuuFNLUiyQwpRNv1m9VTSs/ (last visited Mar. 20, 2025) (on file with author).

47 Authorizing mechanisms for CBJW programs historically have been by administrative order or the office of legal services innovation regulatory sandbox in Utah. *Regulatory Models*, *supra* note 41.

48 For more information about these design choices, see *US Community-Based Justice Worker Programs Information Chart*, *supra* note 46.

49 For a comprehensive examination on the inaction of state bars, see Ralph Baxter, *Dereliction of Duty: State-Bar Inaction in Response to America’s Access-to-Justice Crisis*, 132 Yale L.J.F. 228 (2022).

50
*Id.* at 238 (quoting Stephen P. Younger, *The Pitfalls and False Promises of Nonlawyer Ownership of Law*
*Firms*, 132 Yale L.J.F. 259, 261 (2022)).

51 Balser et al., *supra* note 21, at 87, 104, 106–07.

52
*Id.*

53
*Id.* at 107–08.

54 Houlberg & Drobinske, *supra* note 42, at 11; *Utah Supreme Court to Extend Regulatory Sandbox to Seven Years*, Utah Cts. (May 3, 2021), https://legacy.utcourts.gov/utc/news/2021/05/03/utah-supreme-court-to-extend-regulatory-sandbox-to-seven-years/ [https://perma.cc/BW44-X2ZJ].

55 Balser et al., *supra* note 21, at 109.

56 These models are the Domestic Violence Legal Advocate Initiative in Arizona, the Housing Stability Legal Advocate Initiative in Arizona, the Housing Stability Legal Advocate Initiative in Utah, and the Medical Debt Legal Advocate Initiative in Utah. For more information about these Initiatives, see *Community Legal Education*, Innovation for Just., https://www.innovation4justice.org/education/community [https://perma.cc/J9D4-FM87], (last visited Mar. 20, 2025); *US Community-Based Justice Worker Programs Information Chart*, *supra* note 46.

57
*West Valley Vision: A New Model for Partnering with and Supporting Our Community.*, Univ. of Utah (Mar. 4, 2022), https://attheu.utah.edu/facultystaff/west-valley-vision/ [https://perma.cc/JU6E-GDRC].

58
*Id.*

59
*See The Pillars of Strategy 2025*, Univ. of Utah Health: Good Notes (Mar. 1, 2023), https://uofuhealth.utah.edu/notes/2023/03/strategy-2025-update-directed-steps-2023 [https://perma.cc/E8X2-K7FG].

60
*See* Univ. of Utah Health, Strategy 2025, at 18 (2024), https://uofuhealth.utah.edu/sites/g/files/zrelqx386/files/media/documents/2024/Strategy%20Refresh.2024%284b%29.pdf.

61 Census Reporter, *West Valley City, UT*, http://censusreporter.org/profiles/16000US4983470-west-valley-city-ut/, (last visited April 7, 2025). 2023 Census data shows that only 39% of the population of West Valley City is White, while 44% is Hispanic, 4% is Asian, 4% is Pacific Islander, 4% is 2+ races, 2% is Black, and 2% is Native. *Id.* In contrast, 65% of people in Salt Lake City are white. Census Reporter, *Salt Lake City, UT*, http://censusreporter.org/profiles/16000US4967000-salt-lake-city-ut/ (last visited, April 7, 2025).

62
*Id. See also* Census Reporter, *West Jordan, UT*, http://censusreporter.org/profiles/16000US4982950-west-jordan-ut/ (last visited April 7, 2025); Census Reporter, *Magna, UT*, http://censusreporter.org/profiles/16000US4947290-magna-ut/ (last visited April 7, 2025); Census Reporter, *Taylorsville, UT*, http://censusreporter.org/profiles/16000US4975360-taylorsville-ut/ (last accessed April 7, 2025). The exact number, as of the 2023 Census, is 374,930. Taylorsville has a population of 59,010, Kearns has a population of 37,058, West Jordan has a population of 114,908, Magna has a population of 29,488, and West Valley City has a population of 134,466. *Id.*

63 Census Reporter, *Utah*, http://censusreporter.org/profiles/04000US49-utah/ (last visited April 7, 2025) (the population of Utah is 3,417,734; 75% of the population of Utah is white, 16% is Hispanic, 4% is 2+ ethnicities, 2% is Asian, and only 1% are Black, Native, and Pacific Islander).

64 A cornerstone of CPAR is a commitment to “no research on us, without us,” widening who is considered a researcher beyond the traditional academic setting. Michelle Fine et al., *Critical Participatory Action Research: Methods and Praxis for Intersectional Knowledge Production*, 68 J. Counseling Psych. 344, 345 (2021). CPAR engages communities in an effort to not only document, but also “challenge and transform conditions of social justice.” Clara E. Hill & Sarah Knox, *Series Forward* to Michelle Fine & María Elena Torre, Essentials of Critical Participatory Action Research, at x (2021).

65
*See generally supra* notes 62 and 63.

66 Fine et al., *supra* note 64, at 354–55.

67 Balser et al., *supra* note 21, at 71–74.

68
*Id.* at 98. More information about the methodology for this study can be found in Appendix A.

69 For more information about interview findings, see Cayley Balser, Stacy Rupprecht Jane & Alesia Ash, Innovation for Just., Embedding Regulatory Reform-Based Civil Justice Problem Solving in Patient Care 20–42 (2023), bit.ly/i4J22UHealth. For information about the methodology of the interviews and the questions asked, see *id.* at 12–13, 94–99. For information about interview methods, see *infra* Appendix A.

70 This justice needs survey was developed through a landscape analysis of current justice needs surveys and a comparison to the questions that U of U Health is already asking about patient SDOH. For more information about the development of this justice needs survey, see Balser, Jane, & Ash *supra* note 69. at 13–24.

71 Justice Needs Survey of Nineteen West Valley Community Members (data on file with authors) [hereinafter Justice Needs Survey].

72
*Id.*

73 Zoom Interviews with Health Care Providers in Utah (Fall 2022) [hereinafter Interviews with Health Care Providers] (transcripts on file with author).

74
*Id.*

75
*Id.*

76
*Id.*

77
*Id.*

78
*Id.*

79
*Id.*

80
*Id.*

81
*Id.*

82
*Id.*

83
*Id.*

84
*Id.*

85 Zoom Interviews with Community-Based Organization Staff in Utah (Fall 2022) [hereinafter Interviews with Community-Based Organization Staff] (transcripts on file with author).

86
*Id.*

87
*Id.*; Interviews with Health Care Providers, *supra* note 73.

88 Interviews with Community-Based Organization Staff, *supra* note 85.

89
*Id.*

90
*Id.*

91
*Id.*

92
*Id.*

93
*Id.*

94
*Id.*

95
*Id.*

96
*Id.*

97 Zoom Interviews with Care Managers in Utah (Fall 2022) [hereinafter Interviews with Care Managers] (transcripts on file with author).

98 Interviews with Community-Based Organization Staff, *supra* note 85.

99 In this section of the justice needs survey, three questions were asked: I) “in the past two years, did you experience a problem that involved an attorney, a lawsuit, a court, or a judge?”; II)“in the past two years, have you had a problem you thought might go to court?”; and III) “in the past two years, did you seek legal help for a problem?”

100 Justice Needs Survey, *supra* note 71.

101
*Id.*

102
*Id.*

103 Zoom Interviews with West Valley Community Members (Fall 2022) [hereinafter Interviews with Community Members] (transcripts on file with author).

104
*Id.*

105
*Id.*

106
*Id.*

107
*Id.*

108
*Id.*

109
*Id.*

110
*Id.*

111
*Id.*

112 Interviews with Community-Based Organization Staff, *supra* note 85.

113 Interviews with Health Care Providers, *supra* note 73.

114 For more information about trauma-informed care, see Cayley Balser, *Trauma-Informed Practices at Innovation for Justice (i4J)*, Innovation for Just., https://www.innovation4justice.org/publications/blog/trauma-informed-practices-at-i4j [https://perma.cc/VB93-ZTY5] (last visited Mar. 20, 2025).

115
*Id.*

116 Interviews with Health Care Providers, *supra* note 73.

117 Interviews with Care Managers, *supra* note 97.

118 Interviews with Health Care Providers, *supra* note 73; Interviews with Community-Based Organization Staff, *supra* note 85; *see also* Cayley Balser & Antonio M. Coronado, *Power-Conscious Legal Work: Building a Roadmap for Rural Access to Justice Through Trust, Accountability, & Trauma-Informed Practices*, 41 Alaska L. Rev. 146, 156–61 (2025) (explaining the concept of retraumatization and offering suggestions for how trauma-informed practices can be implemented in legal settings).

119 Interviews with Health Care Providers, *supra* note 73; Interviews with Community-Based Organization Staff, *supra* note 85.

120
*Supra* note 119.

121 Interviews with Health Care Providers, *supra* note 73.

122
*Id.*

123
*See* Balser & Coronado, *supra* note 118, Section V.

124
*Id.*

125 Zoom Interviews with Health Care System Actors in Utah (Fall 2022) [hereinafter Interviews with Health Care System Actors] (transcripts on file with author).

126 Interviews with Health Care Providers, *supra* note 73.

127 Interviews with Community-Based Organization Staff, *supra* note 85.

128
*Id.*

129
*Id.*

130
*Id.*; Zoom Interviews with Justice System Actors in Utah (Fall 2022) [hereinafter Interviews with Justice System Actors] (transcripts on file with author).

131 Interviews with Justice System Actors, *supra* note 130.

132 Interviews with Health Care Providers, *supra* note 73.

133
*Id.*

134
*Id.*

135
*Id.*

136 Interviews with Justice System Actors, *supra* note 130.

137
*Id.*

138 Zoom Interviews with Court System Actors in Utah (Fall 2022) [hereinafter Interviews with Court System Actors] (transcripts on file with author).

139 Interviews with Justice System Actors, *supra* note 130.

140
*Id.*

141 Interviews with Court System Actors, *supra* note 138.

142 Interviews with Justice System Actors, *supra* note 130.

143 Zoom Interviews with Community Health Workers in Utah (Fall 2022) [hereinafter Interviews with Community Health Workers] (transcripts on file with author).

144
*Id.*

145 Interviews with Community-Based Organization Staff, *supra* note 85.

146 Interviews with Health Care Providers, *supra* note 73.

147
*Id.*

148
*Id.*

149
*Id.*

150
*Id.*

151
*Id.*

152
*Id.*

153 Interviews with Community Members, *supra* note 103; Justice Needs Survey, *supra* note 71.

154 Interviews with Community-Based Organization Staff, *supra* note 85.

155
*Id.*

156
*Id.*

157
*Id.*

158
*Id.*

159
*Id.*

160
*Id.*

161
*Id.*

162
*Id.*

163
*Id.*

164
*Id.*

165
*Id.*

166
*Id.*

167
*Id.*

168
*Id.*

169
*Id.*

170 For more information about methods used to gather feedback about the proposed service model, see *infra* Appendix B.

171 Zoom Prototype Feedback Interviews with Healthcare Providers in Utah (Fall 2022) [hereinafter Feedback Interviews with Healthcare Providers] (transcripts on file with author); Zoom Prototype Feedback Interviews with Community-Based Organization Staff in Utah (Fall 2022) [hereinafter Feedback Interviews with Community-Based Organization Staff] (transcripts on file with author).

172 Prototype Feedback Surveys from 69 West Valley Community Members [hereinafter Feedback Surveys from Community Members] (data on file with author).

173
*Id.*

174 Interviews with Community-Based Organization Staff, *supra* note 85; Interviews with Health Care Providers, *supra* note 73.

175 This research team has previously highlighted the potentially high hurdles of LPP certification in Utah: “[a]n applicant for LPP certification [in Utah] must have either (1) an associate’s degree in paralegal studies, (2) a bachelor’s degree in paralegal studies, or (3) a Master of Legal Studies degree. If an applicant does not have any of these educational backgrounds, they may apply if they are a Certified Paralegal, Professional Paralegal, or Registered Paralegal with a credential from authorized agencies. In addition to an educational or professional background, applicants must also have 1,500 hours of law-related experience within the three years prior to application. If they are seeking the family law endorsement, they must complete five hundred of those hours in family-law-related matters. If they are seeking debt collection or landlord-tenant endorsement, they must have one hundred hours in debt collection or forcible entry experience. Applicants must also take and pass an exam. Depending on existing educational background, the cost of LPP certification can be between $600 and $10,000. After applicants are endorsed, active licensing fees are about $220 per year.” Antonio M. Coronado, Rachel Crisler, Cayley Balser & Stacy Rupprecht Jane, *Re-Regulating Justice: Realizing Housing Stability Through Community Legal Advocacy*, 32 J. Affordable Hous. & Cmty. Dev. L. 393, 413 (2024) (citing Houlberg & Drobinske, *supra* note 42, at 50, 74–75 (2022)).

176 Feedback Interviews with Community-Based Organization Staff, *supra* note 171.

177
*Id.*

178
*Id.*; Feedback Interviews with Health Care Providers, *supra* note 171.

179 Sources cited *supra* note 178.

180 Sources cited *supra* note 178.

181 Work on this project continued in Spring 2023. For further information on the ongoing design work, see Cayley Balser & Stacy Rupprecht Jane, Innovation for Just., Mapping the Community Justice Worker Service Model: A Case Study with the University of Utah Population Health Center (2023), https://bit.ly/i4J23PopHealth.

182
*Id.*

183
*Id.* at 4.

184 Vivek Maru, Lyttelton Braima & Gibrill Jalloh, *Squeezing Justice Out of a Broken System: Community Paralegals in Sierra Leone*, *in* Community Paralegals and the Pursuit of Justice 210, 231, 238 (Vivek Maru & Varun Gauri eds., 2018); Anuradha Joshi, Marta Schaaf & Dina Zayed, *The Use of Legal Empowerment to Improve Access to Quality Health Services: A Scoping Review*, 21 Int’l J. Equity Health art. no. 136, at 2 (2022).

185
*See, e.g.*, Maru, Braima & Jalloh, *supra* note 184, at 211, 223–229 (discussing the emergence of community paralegals in Sierra Leone “after the end of an eleven-year civil war as a way of providing basic access to justice and repairing the ties between citizens and state”).

186 Joshi, Schaaf & Zayed, *supra* note 184, at 2.

187
*Id.* at 4.

188
*See* Bård Andreassen, *The Right to Development and Legal Empowerment of the Poor*, Bangladesh Dev. Stud., Mar.-June 2010, at 311, 312 (discussing the 2005 creation of the Commission by the U.N. Development Programme) (citing *Concept Paper for High Level Commission on Legal Empowerment of the Poor: Poverty Reduction through Improved Asset Security, Formalisation of Property Rights and the Rule of Law* (Sept. 6, 2005), [https://web.archive.org/web/20050927114248/http://www.undp.org/legalempowerment/Concept_Paper.doc]). The findings of the Commission were presented in a two-volume report in 2008. *See generally* 1 Comm’n on Legal Empowerment of the Poor, Making the Law Work for Everyone: Report of the Commission on Legal Empowerment of the Poor (2008); 2 Comm’n on Legal Empowerment of the Poor, Making the Law Work for Everyone: Working Group Reports, U. N. Sales No. 08.III.B.14 (2008).

189 G.A. Res. 63/142, Legal Empowerment of the Poor and Eradication of Poverty (Dec. 11, 2008).

190
*See, e.g.*, *US Legal Empowerment Leadership Course*, N.Y.U. Sch. of L.: Robert & Helen Bernstein Inst. for Hum. Rts., https://www.law.nyu.edu/centers/bernstein-institute/legal-empowerment/US-legal-empowerment (last visited Jan. 26, 2024), [https://perma.cc/DE5A-8MJZ]; *Legal Empowerment*, Nat’l Ctr. for Access to Just., https://ncaj.org/tools-for-justice/legal-empowerment [https://perma.cc/2KBB-KYA7] (last visited Jan. 26, 2024).

191
*See, e.g.*, Jesse Jannetta, Jeremey Travis & Evelyn McCoy, U.S. P’Ship on Mobility from Poverty, Participatory Justice, at iv (2018). “Participatory justice combines existing concepts and interventions into an ambitious model that can be piloted, evaluated, and replicated. By so doing, we seek to collectively create safer communities in which the footprint of the criminal justice system is limited.” *Id.*

192
*See, e.g.*, Himonas & Hubbard, *supra* note 22, at 263 (arguing that the Utah judiciary’s efforts to transform the civil legal system “democratize the rule of law by making an understanding of the law and access to our civil legal system more widely affordable and available”); Dorothy E. Roberts, *Democratizing Criminal Law as an Abolitionist Project*, 111 Nw. U. L. Rev. 1597, 1597 (2017) (arguing that abolishing the “anti-democratic functions [of criminal law enforcement] that subordinate black people politically … is essential to making the United States a truly democratic society”).

193
*Service Impact Area*, Innovation for Just., https://www.innovation4justice.org/research/service [https://perma.cc/P9PF-HJAC] (last visited Dec. 20, 2024).

194
*See supra* note 22 and accompanying text.

195 Rebecca L. Sandefur, *The Impact of Counsel: An Analysis of Empirical Evidence*, 9 Seattle J. for Soc. Just. 51, 83 (2010).

196 Balser & Jane, *supra* note 181, at 7.

197 Sandefur, *supra* note 40, at 723.

198
*See* RyLee Curtis, *Lessons from the Community: Building Trust*, Accelerate Learning Cmty. (Apr. 8, 2024), https://accelerate.uofuhealth.utah.edu/impact/lessons-from-the-community-building-trust [https://perma.cc/D8LW-LT2Q]; *University of Utah & West Valley*, Univ. of Utah West Valley, https://westvalley.utah.edu/ [https://perma.cc/L9HG-YENL] (last visited June 10, 2024).

199 Cayley Balser & Stacy Rupprecht Jane, *The Diverse Landscape of Community-Based Justice Workers*, Inst. for the Advancement of the Am. Legal Sys. (Feb. 22, 2024), https://iaals.du.edu/blog/diverse-landscape-community-based-justice-workers [https://perma.cc/J76K-V2CB].

200
*Supra* note 197 and accompanying text.

201
*A Toolkit for Advancing Data-Driven Unauthorized Practice of Law Reform: Domestic Violence Case Study*, Innovation for Just., https://uplpolicytoolkit.org/ [https://perma.cc/6YHN-2DJN] (last visited June 2, 2025).

202
*See US Community-Based Justice Worker Programs Information Chart*, *supra* note 46; Balser & Jane, *supra* note 199.

203 Balser et al., *supra* note 21, at 97–99.

204 Balser & Jane, *supra* note 199 (offering examples of implementations of each of these credentialing models).

205
*Id.*

206 Rostain & Teufel, *supra* note 39, at 1481.

207 Sharona Hoffman & Andy Podgurski, *The Patient’s Voice: Legal Implications of Patient-Reported Outcome Measures*, 22 Yale J. Health Pol’y L. & Ethics 1, 27 (2023).

208 Urb. Inst., Do No Harm Guide: Crafting Equitable Data Narratives 4–5 (Jonathan Schwabish, Alice Feng & Wesley Jenkins eds., 2024), https://www.urban.org/sites/default/files/2024-02/Do_No_Harm_Guide_Crafting_Equitable_Data_Narratives.pdf.

209 Erin Bradley, *Modernizing Data Collection to Improve Rigor in Research Involving Human Participants*, *in* Urb. Inst., *supra* note 208, at 7; Timi Koyejo, *Elite Omitted Variable Bias: Missing Datasets and the Political Economy of Data Collection*, *in* Urb. Inst., *supra* note 208, at 12; Balser & Coronado, *supra* note 118, at 184–85.

210 Service model blueprinting is not discussed further in this Article due to length. To learn more about service model blueprinting and how to create your own service model blueprint for an innovative MLP, see generally Balser & Jane, *supra* note 181.

211 CHW leadership was involved in the design of this initiative. An asynchronous prototype was created for CHWs but received no responses. This is likely because CHWs are very busy within their roles.

212
*Cf. supra* notes 210–11 and accompanying text.

213 Determination letter on file with authors.

214 Datasets on file with authors; a research brief on this RCT assessment is projected for Summer 2025.

215 Datasets on file with author.

216 Datasets on file with author; a research brief on DVLA service outcomes is projected for Summer 2025.

217 i4J supports its Utah community partners in fulfilling their monthly data collection and reporting requirements, as outlined under the Utah regulatory Sandbox.

218 Utah Code Ann. § 63N-16-206 (West 2024).

219 Datasets on file with authors.

220
*Id.*

221
*Id.*

222
*Id.*

223
*Id.*

224 For further information about other community-based justice worker models in the United States, see Balser & Jane, *supra* note 199.

225 Work on this Initiative continued through the 2022-23 academic year. For more information about the development of a service model blueprint in Spring 2023, see *id.*

226 i4J has successfully proposed, received court authorization, and implemented three other UPL-reform-based legal service models. *See supra* note 21 and accompanying text.

227 Nat’l Ctr. for State Cts., 2023 State of the State Courts – National Survey Analysis 2 (2023), https://www.ncsc.org/__data/assets/pdf_file/0039/96879/2023-SoSC-Analysis-2023.pdf; Lydia Saad, *Retail, Pharmaceutical Industries Slip in Public Esteem*, Gallup (Sept. 13, 2023), https://news.gallup.com/poll/510641/retail-pharmaceutical-industries-slip-public-esteem.aspx?version=print&trk=article-ssr-frontend-pulse_little-text-block.

## Appendix A: Design and Systems Thinking Methodology as Used in this Study

The layered design and systems thinking research methodology employed in the study described in this Article — while incorporating community members as active participants in the design process through the CPAR lens — consists of five distinct community-engaged stages: empathy, define, ideate, prototype, and test.^228^ As applied to this project, the *empathy stage* began with establishing a baseline understanding of West Valley patient experiences through a review of existing justice needs research and semi-structured interviews with key actors within the U of U Health system. The research team built upon this foundation by conceptualizing representative patient experiences and journey mapping^229^ patient experiences for four categories: generally healthy patients, new incidents of care patients, chronically ill patients, and emergent patients. The research team used these journey maps to identify the following touchpoints: where patients interact with the health care system, under what circumstances, and what other issues a patient might be experiencing at that time. The research team used these touchpoints, along with qualitative data from initial system actor meetings, to inform the scripts for semi-structured qualitative interviews with diverse system actors^230^ in West Valley.

Interviews in this project were conducted with two categories of participants: 1) system actors who interface with West Valley patients and 2) community members who live in West Valley who are over eighteen years old, have a household income of less than $70,000, have experienced a civil justice need in the past two years, as determined by a screening survey,^231^ and speak English.^232^ Health care system actors included clinic medical and nursing directors, nurses, care managers, physicians, and community health workers. Other system actors included community-based organization directors and staff, social workers, mental health providers, and legal service providers. Overall, forty-seven individuals participated in thirty-minute semi-structured system actor interviews on Zoom.

In the *Define phase*, the research team adapted Code for America’s methodology to analyze and synthesize qualitative data.^233^ Transcripts and interview notes from all interviews were moved to Miro, an online tool that can be used to, among other things, sort data through the use of virtual sticky notes. Each data point — in this case, sentence or impression — from transcripts and interviewer notes was externalized as a sticky note on Miro. Then, the research team analyzed data through affinity mapping.^234^ This project employed grounded theory methodology within the CPAR framework.^235^ A hallmark of grounded theory methodology is simultaneous data collection and analysis, known as constant comparative analysis.^236^ Codes and categories used in analysis were generated from the data, not pre-identified.^237^ Qualitative data was analyzed using a manual, computer-aided approach. The research team annotated transcripts to identify codes and categories through variations in text font, color, and highlight. Next, data points within categories were clustered on Miro based on their relationship to each other and their categories. After categories were identified and data points were clustered within categories, themes were named.^238^ These themes identified the relationship between the data points within the cluster and the category. Themes were then used to surface insights.^239^ After analysis, the research team mapped the interactions in the system and transitioned into the ideation phase.^240^

As a grounding step between the *Define* and *Ideate* phases, the research team interviewed U of U Health leadership using a theory of change framework consisting of four steps. First, the research team identified three key system actor categories — experts from the community, experts from University of Utah, and experts in best practices — and, based on interview data, identified what their definition of health is. Second, the research team looked across these system actor categories to develop a shared definition of health. Third, the research team used that definition to identify what each system actor category would need to meet that definition of health. Last, the research team identified how each system actor category would define success.^241^ The research team used this framework to ensure that any proposed interventions would be consistent with the overall goals and objectives of the diverse system actors involved in the new West Valley hospital.

Working from the landscape analysis, insights, and theory of change analysis, the research team moved into the *Ideate* phase, identifying opportunity spaces and potential interventions to address the justice-involved social, economic, and health needs of the West Valley community. Next, these potential interventions were sorted based on their feasibility and impact. The research team proposed ten interventions, grounded in data from interviews, that align with U of U Health’s theory of change.^242^ These ten interventions were again evaluated by the research team for feasibility and impact. Four interventions moved forward into assumption testing, with the research team creating brief, community-engaged experiments to test the riskiest assumptions before investing energy and resources into the interventions.^243^ Findings from assumption testing provide an early compass for which interventions to advance into *Prototype* and *Testing* phases. Assumption testing took the form of brief semi-structured interviews and surveys to gather rapid community feedback on feasibility of and desire for engagement with the proposed interventions.^244^ The research team identified the riskiest assumptions for each intervention — focusing on the assumptions which must be true in order for the idea to succeed — and created engagement materials to solicit community feedback.^245^ Based on the feedback gathered in this stage, two service model interventions moved forward to the *Prototyping* phase.^246^ *Prototyping* consisted of creating early-stage, low-fidelity representations of the proposed interventions, which were then tested with representative users. Testing these prototypes allowed the research team to gather information about how representative users think and feel about initial concepts prior to investing time and resources into their creation. Such testing can uncover problems with product-market fit and provide guidance about what design, language, visuals, or general message to avoid or focus on in order to ensure interventions resonate with users. The benefit of this prototyping approach is that a research team can quickly gain an overall view of how the product is received by users and make iterative design decisions before investing significant time or resources into a polished final product. Two interventions were tested with four representative user groups.^247^

## Appendix B: Feedback Testing Methodology for this Study

Five system actor categories were selected for feedback testing, also known as prototype testing, in the design and systems thinking research methodology:^248^ 1) those who would authorize the service models (Authorizing); 2) those who would design the service models (Designing); 3) those who would provide the proposed services (Providing); 4) those who would receive the proposed services (Receiving); and 5) those who would be affected by the proposed services (Affected).^249^ To prototype test with these five system actor categories, both synchronous and asynchronous prototypes were created.^250^ Table 2 demonstrates the participation distribution for prototype testing with these system actor categories.

**Table 2.** Participation distribution for prototype testing by system actor category

System actor category	Number of asynchronous prototype testing participants	Number of synchronous prototype testing participants
*Authorizing*	N/A	5
*Designing*	N/A	4
*Providing*	Students: 14 CHWs: 0	1
*Receiving*	69	N/A
*Affected*	2	2

Four additional prototypes were created as asynchronous surveys and were distributed to system actors and community members for the Providing, Receiving, and Affected categories.^251^ Fourteen students completed one asynchronous prototype survey for the Providing category, sixty-nine Utah community members completed a second asynchronous prototype survey for the Receiving category, and two care managers completed another asynchronous prototype survey for the Affected category. An asynchronous prototype for CHWs was created for the Providing category, but no responses were collected.

